# Scarcity in abundance? Spatial inequalities in Rheumatoid Arthritis in a health system with financial equity

**DOI:** 10.1186/s41927-023-00332-z

**Published:** 2023-07-11

**Authors:** Norman Maldonado, Sandra Camacho, Sergio I. Prada, Andrés Hormaza-Jaramillo, Victoria Soto, William García, Nelcy Paredes, Fabián Cardona

**Affiliations:** 1 PROESA – Research Center on Health Economics and Social Protection, Universidad Icesi, Cali, Colombia; 2 Centro de Investigaciones Clínicas, Fundación Valle del Lili, Cra. 98 # 18-49, 760032 Cali, Colombia; 3 Fundación Valle del Lili, Unidad de Reumatología, Cra. 98 # 18-49, 760032 Cali, Colombia; 4 Asociación Colombiana de Empresas de Medicina Integral (ACEMI), Bogotá, Colombia; 5 IES Salud IPS, Bogotá, Colombia

**Keywords:** Spatial inequalities, Health system, Workforce, Service delivery, Governance, Colombia, Rheumatoid arthritis

## Abstract

**Background:**

This paper estimates spatial inequalities of Rheumatoid Arthritis (RA) in Colombia and explores correlates of those disparities from a health system perspective.

**Methods:**

We apply descriptive epidemiology to healthcare administrative records for estimation of crude and age-standardized prevalences, and health systems thinking for identification of barriers to effective access in RA diagnosis.

**Results:**

The crude and age-standardized RA prevalence for Colombia in 2018 is estimated at 0.43% and 0.36%, respectively. In the contributory regime, the binding constraint is effective access to rheumatologists in rural and sparsely populated areas; this constraint in workforce affects service delivery, and ultimately comes from the lack of a differentiated model for effective provision of healthcare in those areas (governance).

**Conclusions:**

There are opportunities for implementation of public health policies and health system interventions that would lead to a better identification of RA patients and the subsequent more precise estimation of RA prevalence, and most importantly, to reduce exposition to risk factors and accurate diagnosis and treatment of RA patients.

## Background

Rheumatoid Arthritis (RA) is an autoimmune and inflammatory disease, that causes damage to several body systems; besides the commonly known effect on the joints, it can also affect the skin, eyes, lungs, heart and blood vessels [1, p.1050, 2]. RA affects quality of life and is a leading cause of functional disability and, when left untreated, the burden of the disease may even cause premature mortality [3]. Unequal access to treatment might severely exacerbate the effects on functional disability and premature mortality, and from there, it can decrease labor force participation and increase poverty and opportunities for social mobility, for both current and future generations [2].

Geography itself plays a core role in health [4], healthcare [5], and in the definition of health equity [6]. In the particular case of RA, space in the form of location of both the patient and the healthcare provider plays an important role in having effective access to healthcare. For the first one, the functional disability caused by the natural history of the disease might become a critical obstacle for autonomous spatial mobility of the patient. For the second one, accurate healthcare for RA needs constant interaction with specialized workforce (rheumatologists or specifically trained internist) and the bedside evaluation and the physical exam in the diagnostic process [7, Ch.2] play a decisive role in getting to a precise diagnosis. Furthermore, RA has some biomarkers (e.g. Rheumatoid factor, C-Reactive Protein or Erythrocyte Sedimentation Rate) for diagnosis and prognosis but none of those work as a single conclusive biomarker, making the frequent physical interaction between the patient and the specialist even more important.

The purpose of this paper is to estimate spatial inequalities of RA in Colombia and to explore correlates of those inequalities, using a health system perspective. In particular, we use administrative records at the health system level to estimate the prevalence of RA and to identify spatial inequalities at the subnational level (departamentos). In addition, based on the national scope and population coverage of the data, we use health systems thinking [15, 16] for the case of RA to explore correlates of those disparities.

The paper contributes to the literature in several ways. First, it provides estimates of RA crude and age-adjusted prevalence using administrative records, bringing together the epidemiological and clinical part of the health system with the economic and financial side of the system. Second, it stresses the importance of space in RA as a determinant of health and healthcare inequalities. Finally, the paper looks beyond identification of disparities and explores correlates of the inequalities, including both risk factors and elements of the health system.

## Method

### Context

Colombia’s national healthcare system is based on the principles of solidarity and managed competition. Currently it is considered to have achieved universal health coverage, with 96% of its population enrolled in public health insurance. There is a mandatory benefits package defined by law that HMOs must secure for all patients in need; benefits include outpatient, inpatient, emergency care, procedures and medicines. There are two schemes within the system, one financed by employee-employer contributions, and one financed by general taxes that subsidizes the benefits package for people unable to pay contributions.Funds are distributed to HMOs using an age-sex and region adjusted capita. HMOs in both schemes are responsible for contracting healthcare providers (e.g. hospitals, laboratories, outpatient services) and creating a healthcare network based on the needs of its affiliates. People are free to choose their HMOs, and HMOs are free to choose their providers.

For the particular case of RA, Colombia has defined a clinical pathway [8] for a potential patient of RA [9], in line with the international evidence on diagnosis and treatment [10]. It recommends the use of ACR/EULAR as criteria for classification, and referral to rheumatologist for confirmation and initiation of treatment in no more than 12 and 16 weeks. Treat-to-target strategy has proven to have the best results [11]; it requires monthly follow-ups with the specialist, until remission [12] or low disease activity. Thus, timely access to a rheumatologist both in the first visit and the follow-ups are vital for accurate diagnosis, effective treatment and prevention of functional loss.

### Model

We do an observational ecological multiple group study [13, 42] using descriptive epidemiology [14] and health systems thinking [15, 16] to estimate the occurrence of RA in Colombia and to explore correlates of the inequalities in such occurrence. To begin with, we estimate RA prevalence as the measure of disease occurrence. Formally, individual *j* = 1*, . . . , J* belongs to a population of *J* individuals (population at risk), has a set *h*_*j,t*_ of healthcare utilizations in year *t* (e.g. doctor visits, diagnostic imaging, prescription drugs, etc), and a subset ${h}_{j,t}^{RA}\subseteq {h}_{j,t}$ is the set of utilizations directly related to RA. RA prevalence ${P}_{t}^{RA}$ is estimated as the number of individuals with healthcare utilizations directly related to RA (${h}_{j,t}^{RA}\ne \varnothing$), expressed as a proportion of population at risk (Eq. 1)

1 $$P_{t}^{{RA}} = \frac{{\mathop \sum \nolimits_{{j = 1}}^{J} I\left( {h_{{j,t}}^{{RA}} \ne \emptyset } \right)}}{J}$$

where $I(\cdot )$ is the indicator function that equals to 1 if condition (·) is met, and 0 otherwise. Thus, RA prevalence is the number of people with healthcare utilizations directly related to RA in a given year, divided by the total population in that year.

Individual *j* has a set of *R* determinants of health [6] and risk factors ${\left\{{r}_{j,t}\right\}}_{r=1}^{R}$, which, for the case of RA, include mainly age, sex, genetic/inherited traits, smoking, history of live births, early life exposures and obesity [17]. We also include determinants related to the health system using a systems thinking approach [15, 16]. Systems thinking is “*an approach to problem solving that views problems as part of a wider dynamic system*”, where “*one looks at the whole system rather than its individual parts*.” [16] The system thinking approach is used to comprehensively explore binding constraints that might limit identification of cases or effective access to healthcare. These health-system determinants are defined by the building blocks of a health system [18].

Under this framework, health disparities in RA are defined as inequalities or disparities in estimated RA prevalence between two categories/values of the same risk factor (e.g. between age groups), that is ${P}_{r{^{\prime}},t}^{RA}{\ne P}_{r{^{\prime}}{^{\prime}},t}^{RA}$, where ${r}^{^{\prime}}$ and ${r}^{{^{\prime}}{^{\prime}}}$ are two categories of values for the risk factor $r$. We focus on spatial inequalities, defined as differences in RA prevalence at the subnational level (among departamentos), and explore correlates of those differences by linking them to spatial disparities in risk factors and health system’s building blocks. Spatial analysis at the subnational level (departamento) is accurate because aggregation at the country level fails to capture disparities, and aggregation at lower spatial levels such as municipalities breaks down the conformation of the network of healthcare providers by the health insurer in a particular region, specially for provision of specialized healthcare such as a visit to a rheumatologist.

For specific risk factors and determinants, we combine the estimation of core indicators for risk factors with a diagnosis of the six building blocks of Colombia’s health system in RA in order to use systems thinking to comprehensively understand the nature of inequalities in RA prevalence. Regarding risk factors, for age, we calculate Age-Standardized prevalences [19] to remove the effect of age on RA inequalities. To calculate the Age-Standardized prevalence, we multiply the RA prevalence for each age group by the average age structure of the world’s population expected over the next generation from 2000 to 2025, called the standard population weight, published by PAHO [19]. The standard population is reported in the PAHO table up to the 100+ age group, but we report the 80+ category by multiplying every age-specific rate in the 80–100+ interval with its respective five-year population weight. We also calculate the aging index at the subnational level [20]. For the other risk factors, we estimate the composition of population by sex, smoking prevalence, history of live births and obesity at the subnational level. We do not include early life exposures and genetic/inherited traits because, to our knowledge, they are unobservable in the sense that there are no administrative records or surveys with data at the subnational level for them. As for the health system, we consider the six building blocks of a health system, namely, (i) service delivery, (ii) health workforce, (iii) information, (iv) medical products, vaccines and technologies, (v) financing, and (vi) leadership and governance [18]. Finally, we use a linear regression model [21] for exploratory analysis on the joint correlation between risk factors, health system variables and spatial disparities in RA prevalence.

### Data

The core dataset is the suficiencia database, which records all healthcare utilizations of every individual insured by the population covered with public insurance. The suficiencia database is used to define the pure premium of the public health insurance, and the Ministry of Health applies a set of validation criteria for inclusion of submitted records in the dataset; both are strong incentives for good-quality data [22].

We limit the analysis to people enrolled in the contributory regime in 2018. RA patients are defined as individuals with at least one healthcare utilization directly related to RA, either via diagnosis using the International Classification of Diseases (ICD-10) or via Classification of drugs using the Anatomic Therapeutic Chemical (ATC) Classification; to control for suspicious but not confirmed cases, we do the analysis also for patients with at least two healthcare utilizations. The ICD codes included are M058, M059, M060, M068 y M069, and the ATC codes included are the ones used by the Colombia’s drug administration agency (Invima) for the following drugs: Abatacept, Adalimumab, Baricitinib, Certolizumab, Etanercept, Golimumab, Infliximab, Leflunomida, Metotrexate, Rituximab, Sulfasalazina, Tocilizumab, Tofacitinib, Azatioprina, Ciclofosfamida and Micofenolato.

We also use microdata from the nationally representative surveys used as official sources of information for each risk factor. Thus, smoking prevalence by departamento is estimated using the National Quality of Life Survey (Encuesta de Calidad de Vida - ECV) in 2018; for live births, we use the Demographic and Health Survey (Encuesta Nacional de Demografía y Salud - ENDS) in 2015; for obesity, we use the National Survey on Nutritional Situation (Encuesta Nacional de situación Nutricional - ENSIN) in 2015; finally, we used data from the 2018 Colombia’s Census of Population and Housing to calculate variables on population’s ageing at the subnational level, namely, population older than 59, the old-age dependency ratio and the aging index.

For health system analysis we combine both quantitative and qualitative information on the health system’s building blocks. Service delivery is analyzed using the utilization of healthcare in the Suficiencia dataset, as well as the number of prescriptions and patients from MIPRES for 2018. For health workforce, we used the registry of rheumatologists’ location collected by the Asociación Colombiana de Reumatología (National Association of Rheumatologists). For information, we identified and reviewed all the sources of information that are being used in the health system to identify RA patients; this was used for both external validation of our estimates and for the systems-thinking analysis in the information building block. For medical products, vaccines and technologies, we look at the benefits package and the coverage of technologies provided by the public health system. For financing, we look at the benefits plan in the public health insurance. Finally, for leadership and governance, we look at the institutional structure that the health system has for provision of healthcare for RA patients.

All data used for the analysis is secondary data either from national surveys or administrative records; therefore, institutional review board or ethics committee approval is not required for this type of study.

## Results

### Health outcomes and disparities

Table 1 shows that the studied population is 16.9 million people (33.8% of Colombia’s population). In this exposed population, under the criteria of at least one healthcare utilization directly related to RA, the number of RA patients is 87,145 and the estimated RA prevalence is 0.51%; under the criteria of at least two healthcare utilizations, the number of RA patients is estimated at 73,168 and the estimated RA prevalence is 0.43%. Since the criteria of at least two healthcare utilizations is more precise in identifying RA patients, from now on we limit the analysis only to those patients. By sex, female prevalence is 4.49 times that of male, and there is an increasing gradient of prevalence over age. Finally, when adjusted by age, RA prevalence goes down to 0.36%.

**Table 1:** Estimated prevalence for Rheumatoid Arthritis (Colombia, 2018)

Group		Population	Patients		Prevalence		A-S prevalence	
			≥ 1	≥ 2	≥ 1 (%)	≥ 2 (%)	≥ 1 (%)	≥ 2 (%)
Total		16,907,798	87,145	73,618	0.5154	0.4354	0.4361	0.3683
Sex								
	Female(F)	8,836,742	71,936	61,182	0.8141	0.6924	0.6593	0.5607
	Male (M)	8,071,056	15,209	12,436	0.1884	0.1541	0.1690	0.1379
Age	0–19	4,411,946	1,087	687	0.0246	0.0156	0.0082	0.0051
	20–39	5,729,805	10,737	8,811	0.1874	0.1538	0.0169	0.0133
	40–59	4,272,424	36,185	31,019	0.8469	0.726	0.0568	0.0466
	60–79	2,112,652	34,192	29,261	1.6184	1.385	0.1855	0.1589
	80+	377,563	4,944	3,840	1.3095	1.017	0.1687	0.1443

Data from Ministerio de Salud y Protección Social de Colombia, Suficiencia dataset. population refers to exposed individuals, defined as individuals enrolled in mandatory public health insurance in the contributory regime. ≥ 1 refers to patients with at least one utilization of healthcare directly related to Rheumatoid Arthritis (RA), and ≥2 refers to those with at least two of those utilizations. Age-Standardized (AS) Prevalence calculated following the method and standard population defined in [19].

Table 2 shows the estimates at the subnational level (departamentos). Among departamentos population coverage varies from 0.3% to 68%. The population under study (contributory regime) is more concentrated (60%) than total population (38%) in 3 main and economically wealthy regions: Bogotá (Colombia’s capital), Antioquia and Valle del Cauca. Most estimates of RA prevalence range between 0.1% (Vichada) and 0.68% (Caldas), and such variation evidences the important disparities in disease occurrence as well as in the local health systems’ capacity to diagnose patients; also, those disparities hold when adjusting for differences in age structure.

**Table 2:** Estimated RA prevalence by Departamento (≈ State) (Colombia, 2018)

Population		Patients		Prevalence		A-S prevalence	
		1	2	1 (%)	2 (%)	1 (%)	2 (%)
Amazonas	13,542	81	64	0.5981	0.4726	0.6121	0.4712
Antioquia	3,007,324	17,380	15,613	0.5779	0.5192	0.4740	0.4262
Arauca	20,258	48	36	0.2369	0.1777	0.2679	0.2118
A.San Andrés, P Y C	39,316	60	48	0.1526	0.1221	0.1334	0.1054
Atlántico	857,561	2,847	2,417	0.3320	0.2818	0.2914	0.2475
Bogotá, D.C.	5,036,963	26,470	22,376	0.5255	0.4442	0.4544	0.3837
Bolívar	475,277	1,644	1,377	0.3459	0.2897	0.3187	0.2668
Boyacá	227,287	1,691	1,263	0.7440	0.5557	0.6201	0.4620
Caldas	363,846	2,906	2,472	0.7987	0.6794	0.5834	0.4958
Caquetá	40,225	167	134	0.4152	0.3331	0.4112	0.3280
Casanare	61,997	131	100	0.2113	0.1613	0.2463	0.1911
Cauca	201,971	1,215	1,003	0.6016	0.4966	0.4921	0.4072
Cesar	239,821	1,136	887	0.4737	0.3699	0.5012	0.3923
Chocó	29,809	68	63	0.2281	0.2113	0.2103	0.1939
Córdoba	247,873	1,551	1,286	0.6257	0.5188	0.5576	0.4622
Cundinamarca	1,093,278	5,155	4,202	0.4715	0.3843	0.4507	0.3672
Guainía	143	-	-	0.0000	0	0.0000	0.0000
Guaviare	10,011	25	19	0.2497	0.1898	0.3236	0.2692
Huila	157,526	674	476	0.4279	0.3022	0.3761	0.2665
La Guajira	97,734	208	164	0.2128	0.1678	0.2252	0.1796
Magdalena	240,492	1,043	887	0.4337	0.3688	0.4197	0.3577
Meta	208,264	562	454	0.2698	0.218	0.2630	0.2128
Nariño	126,323	1,428	1,082	1.1304	0.8565	0.8333	0.6340
Norte De Santander	217,531	1,128	930	0.5185	0.4275	0.4133	0.3368
Putumayo	10,399	40	27	0.3847	0.2596	0.3443	0.2318
Quindio	175,347	1,177	908	0.6712	0.5178	0.4790	0.3747
Risaralda	390,168	2,641	2,233	0.6769	0.5723	0.5282	0.4473
Santander	795,812	2,999	2,471	0.3768	0.3105	0.3276	0.2700
Sucre	97,236	461	371	0.4741	0.3815	0.4032	0.3271
Tolima	329,664	1,342	1,106	0.4071	0.3355	0.3257	0.2682
Valle Del Cauca	2,086,961	10,856	9,142	0.5202	0.4381	0.4004	0.3371
Vaupés	1,562	1	1	0.0640	0.064	0.0316	0.0316
Vichada	6,277	10	6	0.1593	0.0956	0.2045	0.1223

Data from Ministerio de Salud y Protección Social de Colombia, Suficiencia dataset. population refers to exposed individuals, defined as individuals enrolled in mandatory public health insurance in the contributory regime. ≥ 1 refers to patients with at least one utilization of healthcare directly related to rheumatoid disease, and ≥ 2 refers to those with at least two of those utilizations. Age groups defined as the ones used for calculation of the premium of the public health insurance (Unidad de Pago por Capitación - UPC).

These disparities in RA prevalence were grouped in three different categories to identify patterns of RA prevalence and determinants within each category. The classification was done by sorting departamentos by A-S RA prevalence and using as thresholds the highest change in prevalence between consecutive pairs of departamentos. The first group was defined as departamentos with low A-S prevalence (<0.24%) including Putumayo, Meta, Arauca, Chocó, Casanare, La Guajira, Vichada, A. San Andrés y Vaupés; second, departamentos with medium prevalence (<0.4%) including Cesar, Bogotá, Quindío, Cundinamarca, Magdalena, Valle del Cauca, Norte de Santander, Caquetá, Sucre, Santander, Guaviare, Tolima, Bolívar, Huila, Atlántico, Putumayo; third, departamentos with high prevalence (>0.40%) including Nariño, Caldas, Amazonas, Córdoba, Boyacá, Risaralda, Antioquia, Cauca. Also, Guainía and Vaupés have the lowest values for population exposed, making the estimates for prevalence unreliable; for that reason, we exclude them from the analysis thereon. The median value of the remaining departamentos is around 0.3%, a reasonable value of RA prevalence in South America [24]. With the exception of Meta, the group of low prevalence corresponds to rural departamentos with sparsely populated areas; this is relevant because despite of having population-wide administrative records, most of the people in these departamentos are enrolled in the subsidized regime and therefore the data does not accurately represent the situation of RA in those locations. This grouping is relevant because it allows to more precisely look beyond the RA prevalence in terms of exploring correlates of those disparities in quantifiable indicators of RA risk factors and health system’s building blocks; These indicators are shown in Tables 3 and 4, respectively, sorted by prevalence.

**Table 3:** RA Quantifiable risk factors related to population (Colombia, 2018)

Departamento	A-S prevalence (%)	Aging index (%)	Female (%)	Smoking (%)	W. No children	Adult obesity (%)
					(age 30–49) (%)	
High						
Nariño	0.63	38.3	49.81	5.78	8.76	16.96
Caldas	0.50	57.07	51.10	8.6	16.25	16.41
Amazonas	0.47	12.45	49.91	5.01	5.28	27.09
Córdoba	0.46	29.28	49.93	3.94	10.45	17.87
Boyacá	0.46	45.09	49.82	5.28	11.61	16.89
Risaralda	0.45	52.5	51.33	6.72	10.42	16.13
Antioquia	0.43	39	51.12	8.02	15.18	17.63
Cauca	0.41	33.29	49.36	4.25	7.75	17.87
Medium high						
Cesar	0.39	19.36	50.07	4.13	6.17	17.41
Bogotá, D.C.	0.38	40.56	51.54	7.93	14.07	16.73
Quindio	0.37	63.2	50.90	6.98	11.91	18.02
Cundinamarca	0.37	36.6	50.17	6.24	9.64	17.14
Magdalena	0.36	23.13	49.43	4.71	8.27	21.5
Valle Del Cauca	0.34	40.58	51.59	6.4	14.65	22.98
Norte De Santander	0.34	29.33	50.47	5.25	7.63	17.6
Caquetá	0.33	19.43	50.01	3.2	4.78	21.62
Medium low						
Sucre	0.33	29.55	49.34	5.68	10.05	16.7
Santander	0.27	40.17	50.56	5.83	11.69	15.97
Guaviare	0.27	14.7	47.96	5.55	3.85	22.84
Tolima	0.27	47.39	50.07	6.82	11.66	18.96
Bolívar	0.27	26.83	49.99	4.15	8.55	19.72
Huila	0.27	27.37	49.84	4.16	9.67	19.73
Atlántico	0.25	29.06	50.56	3.79	13.18	22.94
Putumayo	0.23	20.56	49.54	1.92	7.93	24.14
Low						
Meta	0.21	26.02	50.14	6.27	6.53	23.58
Arauca	0.21	19.2	49.84	2.89	7.32	20.8
Chocó	0.19	15.92	49.83	4.13	6.15	22.8
Casanare	0.19	18.18	49.39	5.2	5.47	19.72
La Guajira	0.18	13.29	50.49	4.41	11.27	19.48
Vichada	0.12	8.14	49.50	2.92	5.59	26.87
A.San Andrés, P Y C	0.11	30.17	51.55	3.59	10.48	28.38
Vaupés	0.03	7.62	50.01	12.81	7.46	10.93
Guainía	-	8.98	48.62	7.55	3.38	21.88

Data from Ministerio de Salud y Protección Social de Colombia, Suficiencia dataset. population refers to exposed individuals, defined as individuals enrolled in mandatory public health insurance in the contributory regime. *≥* 2 refers to patients with at least two of utilizations directly related to Rheumatoid Arthritis (RA). Age-standardized prevalence calculated following the method and standard population defined in [19].

**Table 4:** RA Quantifiable risk factors related to health systems (Colombia, 2018)

Departamento	A-S prevalence (≥ 2) (%)	Contributory regime (%)	# Utilizations (per patient/year)	Rheumatologists (Per 100,000 people)
High				
Nariño	0.63	19.63	71.02	2.37
Caldas	0.50	53.26	93.59	0.82
Amazonas	0.47	24.35	40.19	0
Córdoba	0.46	18.78	67.75	1.61
Boyacá	0.46	40.39	85.28	0.88
Risaralda	0.45	60.19	80.06	1.54
Antioquia	0.43	61.49	100	1.53
Cauca	0.41	23.62	76.24	0.5
Medium high				
Cesar	0.39	28.49	85.62	0.83
Bogotá, D.C.	0.38	82.69	83.55	1.77
Quindio	0.37	54.46	76.86	0.57
Cundinamarca	0.37	60.67	80.07	0.46
Magdalena	0.36	28.1	87.49	1.25
Valle Del Cauca	0.34	59.11	86.3	1.2
Norte De Santander	0.34	32.81	60.77	0.92
Caquetá	0.33	21.54	53.52	0
Medium low				
Sucre	0.33	16.39	72.71	0
Santander	0.27	53.49	75.06	1.26
Guaviare	0.27	23.54	69	0
Tolima	0.27	41.68	70.02	0.3
Bolívar	0.27	30.71	72.63	1.47
Huila	0.27	31.59	73.4	3.81
Atlántico	0.25	44.84	82.12	1.63
Putumayo	0.23	15.55	65.89	0
Low				
Meta	0.21	46.13	74.01	0.96
Arauca	0.21	20.64	31.22	0
Chocó	0.19	12.83	51.27	0
Casanare	0.19	42.05	49.75	0
La Guajira	0.18	16.87	51.79	0
Vichada	0.12	12	28.17	0
A.San Andrés, P Y C	0.11	74.81	39.46	0
Vaupés	0.03	11.58	7	0
Guainía	0.00	11.69	0	0

Data from Ministerio de Salud y Protección Social de Colombia, Suficiencia dataset. population refers to exposed individuals, defined as individuals enrolled in mandatory public health insurance in the contributory regime. ≥ 1 refers to patients with at least one utilization of healthcare directly related to Rheumatoid Arthritis (RA), and≥2 refers to those with at least two of those utilizations. Age-standardized prevalence calculated following the method and standard population defined in [19].

Regarding risk factors, Table 3 shows that the aging index tends to be higher in departamentos with high RA prevalence. Also, the relative difference in the average of aging index between departamentos with high and medium prevalence is lower than that between departamentos of medium and low prevalence, suggesting that departamentos with low RA prevalences tend to have younger populations and lower affiliation to the contributory regime. Sex composition does not seem to play a role in disparities in RA prevalence because its spatial variation is negligible. Regarding the history of live births, there is wide heterogeneity, and such heterogeneity is higher in departamentos with high RA prevalence. Prevalence of smoking is higher in departamentos with high RA prevalence, and it smoothly decreases when moving to lower categories of RA prevalence. Regarding obesity, the connection with RA prevalence seems to be counterintuitive because departamentos with high RA prevalence have lower prevalence of adult obesity.

### Health system

#### Medical products, vaccines and technologies

Colombia has the same generous health benefit plan (PBS) for all population [25], and when prescribed technologies are not included, patients can access them using a system for records of prescriptions out of the benefit plan (MIPRES). For the specific case of RA, the PBS includes all medications and procedures in the clinical pathway [26]; moreover, it includes almost all treatments available in developed countries, such as conventional synthetic DMARD (Disease-Modifying Antirheumatic Drug), targeted synthetic DMARD and biologic DMARD. Moreover, we do not identify any barrier to effective access to medications because the Health Management Organizations (HMOs) can deliver the medication all over the country, and the network of prescription delivery services has a wide coverage of municipalities.

#### Financing

Colombia’s health system has funding coming from general taxes and payroll contributions, and the funding is spent in providing the PBS for all population [25]. For RA, the inclusion of all technologies provides financial protection from catastrophic risk expenditure. Despite that scope, the contributory regime has a potentially binding constraint related to extended Out-Of-Pocket expenditure [27], because the PBS for that regime does not cover transport to reach healthcare providers, which might become particularly expensive in rural and sparsely populated areas.

#### Information

Regarding information, the country has administrative records on healthcare utilization for RA via RIPS, suficiencia and most importantly from CAC, that records not only morbidity in incidence and prevalence, but also health risk management via monitoring of the clinical pathway for identified and validated RA patients. Furthermore, as shown in Tables 3 and 4, when combining multiple sources of information it is possible to have reliable information on health status, health determinants, and health systems performance.

#### Workforce

The clinical pathway for RA suggests that effective access to rheumatologists is essential for precise diagnosis and the subsequent estimation of prevalence. Table 4 shows that this is not the case in all departamentos: except for Meta, departamentos with low prevalence have zero rheumatologists, showing a clear barrier to healthcare access. There is no universally accepted minimum number of rheumatologists for a country [28]. From an empirical perspective, an overall rate of 1 rheumatologist per 106,838 people in Latin American countries, and for Colombia the rate is 1 per 253,255 [28]; given that Colombia’s population from the 2018 Census of Population was 48,258,494 people, we estimate that the number of people per rheumatologist is 208,111.

#### Service delivery

The binding constraint in workforce translates into a binding constraint in service delivery.Table 4 shows that there is access to general healthcare in departamentos with low prevalence, suggesting that there are no binding restrictions in access to laboratory tests and imaging studies.

This result suggests that the lack or rheumatologists in rural and sparse areas might be leading to underestimation of RA prevalence; furthermore, the binding constraint in workforce translates in limitations on service delivery for screening and accurate diagnosis of RA, meaning that there is an important gap between observed and expected RA patients in those areas.

#### Leadership/governance

Even though there was a pilot of a differentiated model for provision of healthcare in rural areas [29], as of today there is no such model operating at the national scale to guarantee provision of specialized healthcare, as the one required in the clinical pathway for people living in rural and sparsely populated areas. Telemedicine should be an important component of such model [30, 31], in addition to strategies to overcome the safety and economic barriers for spatial mobility of rheumatologists. In general, even though there are strengths on provision of healthcare in RA in technologies, financing and information, this does not necessarily translate into government-level decisions to tackle barriers to healthcare access in departamentos with apparently low-income RA prevalence.

#### Systems thinking

Bringing the health systems building blocks together, the analysis shows that there are binding constraints for effective access to RA diagnosis that are relevant for spatial disparities in RA; in particular, timely access to rheumatologists in rural and sparsely populated areas.

## Discussion

We found an estimated RA prevalence of 0.43% in 2018, important spatial disparities in that prevalence and some risk factors (population aging and smoking) strongly correlated with those disparities. The health systems thinking analysis suggested that the binding constraint for RA in Colombia is workforce in rural and sparse areas, which directly affects service delivery, and the reason behind that seems to be the lack of a healthcare provision model in those areas (governance). When looking at risk factors and elements related to the health system simultaneously, we found that there are opportunities for action on both prevention diagnosis and treatment of RA [32]. Moreover, results of an exploratory linear regression analysis (Table 5) suggests that there is joint significance of the variables involved, and that the explanatory power of these factors reaches about 60%; this suggests that a comprehensive approach tackling the binding constraints for diagnosis and treatment of RA as well as exploiting the full potential of public health intervention on risk factors can significantly reduce disparities in RA.

**Table 5:** Regression analysis (Colombia, 2018)

Variable	RA prevalence
Intercept	− 0.023050606
	(0.0241)
\% population in the contributory regime	− 0.000064**
	(0.00002)
\# healthcare utilizations	1.52711E−09
	(0)
\# Rheumatologists	− 2.65466E−05
	(0.00005)
Aging Index	0.01449***
	(0.00311)
\% Female	0.053714814
	(0.0495)
Smoking prevalence	− 0.011650366
	(0.01533)
\% Women with no children	− 0.000201875
	(0.00014)
Obesity prevalence	− 2.40364E−05
	(0.00009)
\% Population 60+	2.89295E−09
	(0)

There are previous estimates of RA prevalence from other sources such as Cuenta de Alto Costo (CAC), with an RA prevalence of 0.408% and an age-adjusted RA prevalence of 0.395%, other local estimates of 0.52% [23], regional estimates of 0.3% [24] and global estimates of 0.5–1% [2]. The proximity of our estimates with the previous ones seems to externally validate our results; however, previous studies do not specify the age groups or the standard age structure used (CAC), or whether any standardization was done in the estimates [23, 24], and thus they are not directly comparable.

The main limitations of the study are the natural limitations of ecological studies, that is, the loss of information from using aggregate data, the upward bias in correlations from working at the group level rather than at the individual level, the impossibility of doing causal inference, and the risk of the ecological fallacy [42]. Since these limitations come from the nature of the study, it is not possible to overcome them; instead, we have limited the scope of the arguments derived from the data analysis to keep them at the group level. Despite these limitations, the estimates have an important value in the literature because they introduce environmental aspects, which is the main contribution of ecological studies, and they take the analysis of RA prevalence to a health-system level, a level necessary for health policy.

Regarding other potential limitations, the dataset might have measurement error due to its nature of administrative records. However, the asymptotics from population coverage, the incentives for accurate record of data and the validation criteria from the Ministry of health seem to be working in correcting that error, because the proximity of estimated RA prevalence to those from global, regional and local studies suggest that such correction is taking place and estimates are valid. This is an important result, as the suficiencia database connects diagnosis, healthcare provision and costs, opening an opportunity in future research to estimate costs of provision of healthcare for RA and spatial differences in such provision and costs. There might be additional noise from using data on some risk factors for years other than 2018. However, we chose the most reliable sources on those variables with the closest date to 2018, given that such data is not produced every year. Also, those risk factors at the subnational level are not expected to have sharp variations in the short run.

The connection between RA prevalence, risk factors and health system conditions is complex in the sense that it is nonlinear, it has two-way causality, and there are multiple and dynamic interactions among them [33]. Furthermore, not all aspects of each factor and condition can be quantified, and in those that can be, there are multiple ways to measure them, and many of them might have measurement error. For that reason, the analysis of joint significance and correlates of the variability in RA prevalence is only exploratory because we recognize that a linear static regression is an extremely weak representation of the complexity behind those disparities in RA prevalence. Despite that limitation, we find value in bringing together new estimates of RA prevalence with quantifiable measures of most risk factors and some of the health system building blocks; also, the exploratory joint analysis suggests that there are comprehensive actions on risk factors and binding constraints at the health system level can significantly decrease both total RA prevalence and spatial disparities. Examples of these actions are, in the side of risk factors, an accurate implementation of the comprehensive set of interventions to control smoking specified in the Framework Convention on Tobacco Control, and, in the side of provision of healthcare, a comprehensive health workforce strategy that sets up interdisciplinary groups (nurses, general practitioners, specialists) scattered across different spatial locations and effectively support their interaction with telemedicine to improve diagnosis and treatment in rural and sparse areas.

## Conclusions

On risk factors, the analysis highlights the importance of local implementation of effective policies on tobacco control [34, 35] and obesity [36], 37, Part4), taking into account the particular conditions of the country [38]. At the same time, it is urgent to accelerate the adaptation of the health system and social protection systems to accurately respond the to the needs of an aging population [39], especially in the workforce and service delivery building blocks [40, 41]. As a last point, findings related to rural and sparse areas highlight the need of a similar analysis for the population in the subsidized regime, a regime that concentrates the people living in those areas. In terms of health policy, the analysis highlights the need of implementation of public health policies and health system interventions to better identify RA patients, to have a more precise estimation of RA prevalence, and most importantly, to reduce exposition to risk factors and accurate diagnosis and treatment of RA patients. One example of this need is the urgency of a differentiated model for effective provision of healthcare in those areas (governance).

## Data Availability

The datasets generated and/or analysed during the current study are not publicly available due to privacy concerns because it is not deidentified; they are available from the corresponding author on reasonable request.

## Abbreviations

RA: Rheumatoid arthritis
HMOs: Health management organizations
PBS: Benefit plan
MIPRES: System for records of prescriptions out of the benefit plan
DMARD: Disease-modifying antirheumatic drug
ICD-10: International classification of diseases
ATC: Anatomic therapeutic chemical

## References

[1] Barrero LH, Caban-Martinez AJ, Detels R (2015). 89 Musculoskeletal disorders. Oxford textbook of global public health.

[2] Hashimoto T, Gellman MD (2020). Rheumatoid arthritis: psychosocial aspects. Encyclopedia of behavioral medicine.

[3] Uhlig T, Moe RH, Kvien TK (2014). The burden of disease in rheumatoid arthritis. Pharmaco Economics..

[4] Gatrell AC, Elliott SJ (2015). Geographies of health: an introduction.

[5] Andrews GJ, Crooks VA. Geographical perspectives on health care: ideas, disciplines, progress. In: Crooks VA, Andrews GJ (eds) Primary health care: people, practice, place. Ashgate, 2009. Chap. Ch.2.

[6] Solar O, Irwin A. A Conceptual Framework for Action on the Social Determinants of Health. Tech. rep. 2. World Health Organization (WHO), 2010.

[7] Balogh EP, Miller BT, Ball JR (eds) Improving Diagnosis in Health Care. Institute of Medicine, The National Academies of Sciences, Engineering, and Medicine, The National Academies Press, Washington, DC, 2015. 26803862

[8] Lawal AK (2016). What is a clinical pathway? Refinement of an operational definition to identify clinical pathway studies for a Cochrane systematic review. BMC Med.

[9] Minsalud and Colciencias. Guía de Práctica Clínica para la detección temprana, diagnóstico y tratamiento de la artritis reumatoide. Colciencias, MinSalud, 2014.

[10] Liu Shuang (2018). Rheumatoid arthritis: methods and protocols.

[11] Aletaha D, Smolen JS (2018). Diagnosis and management of rheumatoid arthritis: a review. JAMA..

[12] Ma MHY, Scott IC, Kingsley GH, Scott DL (2010). Remission in early rheumatoid arthritis. J Rheumatol..

[13] Jewell NP (2009). Statistics for epidemiology.

[14] Bruce N, Pope D, Stanistreet D. Quantitative methods for health research: a practical interactive guide to epidemiology and statistics. 1st Ed. 2008.

[15] Savigny D, Adam T (2009). Systems thinking for health systems strengthening. Alliance for Health Policy and systems Research.

[16] Johnson JA, Anderson DE, Rossow CC. Health systems thinking: a primer. Jones & Bartlett Learning, 2020.

[17] CDC. Rheumatoid Arthritis (RA). 2022.

[18] WHO. Everybody’s business: strengthening health systems to improve health outcomes. WHO’s framework for action. World Health Organization (WHO), 2007.

[19] PAHO. Age standardized mortality rates per 100,000 population deaths less 70 years, 2009. Tech. rep. Pan American Health Organization (PAHO), 2009.

[20] Morgan LA, Kunkel SR (2007). Aging, society, and the life course.

[21] Stock J, Watson M. Introduction to econometrics. 3rd Ed. Pearson, 2015.

[22] Minsalud. Estudio de suficiencia y de los mecanismos de ajuste del riesgo para el cálculo de la Unidad de Pago por Capitación, recursos para garantizar la financiación de tecnologías en salud y servicios en los regímenes Contributivo y Subsidiado. 2020. Tech. rep. Dirección de Regulación de Beneficios, Costos y Tarifas del Aseguramiento en Salud, Ministerio de Salud y Protección Social (Minsalud), República de Colombia, 2019.

[23] Fernández-Ávila DG, Rincón-Riaño DN, Bernal-Macías S, Gutiérrez-Dávila JM, Rosselli D (2019). Prevalencia de la artritis reumatoide en Colombia según información del Sistema Integral de Información de la Protección Social. Revista Colombiana de Reumatología..

[24] Almutairi K, Nossent J, Preen D, Keen H, Inderjeeth C (2021). The global prevalence of rheumatoid arthritis: a meta-analysis based on a systematic review. Rheumatol Int..

[25] Giedion U, Cañón O. Colombia: The Compulsory Health Plan. Health Benefit Plans in Latin America. Inter-American Development Bank. Social Protection and Health Division, 2014. p. 76–109.

[26] MinSalud. Resolución 5269 Por la cual se actualiza integralmente el Plan de Beneficios en Salud con cargo a la Unidad de Pago por Capitación (UPC). Tech. rep. Ministerio de Salud y Protección Social (MinSalud), República de Colombia, 2017.

[27] Saksena P, Xu K, Elovainino R, Perrot J. Health services utilization and out-of-pocket expenditure at public and private facilities in low-income countries. World Health Report. 2010. 10.1111/j.1365-3156.2011.02894.x 22008480

[28] Fernández-Ávila DG, Patino-Hernandez D, Kowalskii S, Vargas-Caselles A, Sapag AM (2021). Current status of the rheumatologists’ workforce in Latin America: a PANLAR collaborative study. Clin Rheumatol..

[29] MinSalud. Modelo Integral de Atención en Salud – MIAS. Piloto de Implementación en los Departamentos con Poblaciones Dispersas: Departamento de Guainía. Tech. rep. Ministerio de Salud y Protección Social (MinSalud), República de Colombia, 2015.

[30] Rezaian MM, Brent LH, Roshani S, Ziaee M, Sobhani F (2019). Rheumatology care using telemedicine. Telemedicine and e-Health..

[31] Hormaza-Jaramillo A, et al. “Effectiveness of telemedicine compared with standard care for patients with rheumatic diseases: a systematic review”. 2022. 10.1089/tmj.2022.0098 35834601

[32] Jackson MC, Sambo LG (2020). Health systems research and critical systems thinking: the case for partnership. Syst Res Behav Sci..

[33] Sturmberg J, Martin C. Handbook of systems and complexity in health. Springer, 2013.

[34] WHO. WHO framework convention on tobacco control. Tech. rep. World Health Organization (WHO), 2004.

[35] UNDP et al. Investment Case for Tobacco Control in Colombia. The case for scaling-up WHO FCTC implementation. Tech. rep. United Nations Development Program (UNDP), Who Framework Convention on Tobacco Control Secretariat (FCTC), Pan American Health Organization (PAHO), Research Triangle Institute (RTI), 2019.

[36] WHO. Tackling NCDs. ’Best buys’ and other recommended interventions for the prevention and control of noncommunicable diseases. Tech. rep. Technical Report WHO/NMH/NVI/17.9, World Health Organization (WHO), 2017.

[37] Cawley J. Oxford handbook of the social science of obesity. Oxford University Press, 2011.

[38] Allen LN, Pullar J, Wickramasinghe K, Williams J, Foster C (2017). Are WHO "best buys" for non-communicable diseases effective in low-income and lower-middle-income countries? A systematic review”. Lancet Glob Health..

[39] Dexter PR, Miller DK, O Clark D, Weiner M, Harris LS, et al. Preparing for an aging population and improving chronic disease management. In: AMIA Annual symposium proceedings*.* 2010;2010:162–166. PMC3041380 21346961

[40] Dall TM, Gallo PD, Chakrabarti R, West T, Semilla AP (2013). An aging population and growing disease burden will require a large and specialized health care workforce by 2025. Health Affairs..

[41] Ansah JP, Eberlein RL, Love SR, Bautista MA, Thompson JP (2014). Implications of long-term care capacity response policies for an aging population: A simulation analysis. Health Policy..

[42] Boslaugh, S. Encyclopedia of Epidemiology. 2008. ISBN: 978-1-4129-2816-8. SAGE publications.

